# Assessment of Measles Immunity in the Croatian Population: A Retrospective Seroprevalence Study, 2015–2025

**DOI:** 10.3390/vaccines14050393

**Published:** 2026-04-27

**Authors:** Tatjana Vilibić-Čavlek, Klara Barbić, Vesna Višekruna Vučina, Maja Bogdanić, Bernard Kaić

**Affiliations:** 1 WHO National Measles/Rubella Laboratory, Department of Virology, Croatian Institute of Public Health, 10000 Zagreb, Croatia; maja.bogdanic@hzjz.hr; 2 School of Medicine, University of Zagreb, 10000 Zagreb, Croatia; 3 Statistic Concentrator, Harvard University, Cambridge, MA 02139, USA; klarabarbic@college.harvard.edu; 4 Department of Communicable Disease Epidemiology, Croatian Institute of Public Health, 10000 Zagreb, Croatia; vesna.visekruna@hzjz.hr (V.V.V.); bernard.kaic@hzjz.hr (B.K.)

**Keywords:** measles virus, seroprevalence, epidemiology, Croatia

## Abstract

Background/Objectives: Measles is a highly contagious infectious disease. Maintaining a high level of measles immunity in the population is essential due to the extremely high transmissibility of the measles virus (MV). We analyzed the MV seroprevalence in the Croatian population. Methods: A total of 1998 individuals tested consecutively for MV antibodies from 2015 to 2025 inclusive were included. MV IgG antibodies were detected using a commercial ELISA. Results: The overall seroprevalence rate was 75.1%, with significant yearly differences, ranging from 50.0 to 86.5%, and a declining trend since 2023. No differences were observed between sexes (males 71.6%, females 71.9%). The seroprevalence increased with age, from 60.9% in the 1–10 age group to 91.1% in the 61+ age group. Significant geographic differences were found, with higher seropositivity rates in coastal regions compared to continental regions (81.3 and 71.0%, respectively). Settlement type (urban, suburban/rural) was not associated with the MV seroprevalence. The results of the multivariable logistic regression analysis showed that year, age, and region were significantly associated with IgG seropositivity. Each additional calendar year was associated with lower odds of IgG positivity (OR = 0.82), while each additional year of age was associated with higher odds (OR = 1.04). Region was also a significant predictor (OR = 1.65), while settlement was not significantly associated with seropositivity after adjustment for other variables. Conclusions: Croatia has historically maintained high measles coverage, but lower uptake in some regions and age groups may be creating immunity gaps. The lower post-2022 seropositivity underscores significant immunity gaps, particularly among highly susceptible groups that were exposed to outbreaks and tested during that period.

## 1. Introduction

Measles is a highly contagious infectious disease caused by the measles virus (MV; *Morbillivirus hominis* according to the 2024 ICTV classification) of the family *Paramyxoviridae*, genus *Morbillivirus* [1]. The MV comprises a single serotype but 24 different genotypes that belong to eight clades (A–H). Genotyping is used in molecular epidemiological surveillance to identify transmission pathways during outbreak investigations and to monitor the progress of elimination by differentiating between wild-type and vaccine MV strains [2].

MV primarily spreads via respiratory droplets during coughing or sneezing. Infected individuals are contagious from about four days before to four days after the onset of the rash [3]. Transmission occurs year-round, with seasonal peaks often observed in late winter and early spring in temperate climates. Measles remains a leading cause of morbidity and mortality worldwide, despite the availability of an effective vaccine. Outbreaks commonly occur due to immunity gaps, population migrations, and declines in routine immunization coverage [4]. Before the widespread use of the measles vaccine, major outbreaks occurred every 2–3 years and resulted in an estimated 2.6 million deaths annually worldwide during the pre-vaccine era. The introduction of routine immunization has substantially reduced both global incidence and mortality [5].

Measles typically starts with a prodromal phase characterized by fever, malaise, cough, coryza, and conjunctivitis. Pathognomonic Koplik spots may appear on the buccal mucosa before the onset of rash. The exanthematous phase follows, characterized by a generalized maculopapular rash, often coinciding with peak viremia and high fever [6]. While symptoms usually resolve within several days, measles can result in severe complications, including pneumonia, otitis media, acute encephalitis, and, rarely, subacute sclerosing panencephalitis [7]. Complications and mortality occur more frequently in young children, pregnant women, malnourished individuals, and immunocompromised individuals [8].

In 2024, global coverage of the first dose of an MV-containing vaccine was estimated at 84%, remaining slightly below pre-pandemic levels and exhibiting substantial regional heterogeneity. Compared to pre-pandemic 2019 levels, the estimated number of measles cases increased by 8%, while measles-related deaths declined by 11%. This divergence likely reflects a recent shift in the distribution of disease burden toward middle-income countries, where fatality rates are generally lower [9].

In the European Region, the incidence of measles declined steadily from 1997. However, this declining trend was interrupted by a resurgence in 2018 and 2019. Following declines in immunization coverage during the COVID-19 pandemic, measles cases increased markedly again in 2023 and 2024. In 2024, Europe reported the highest number of cases since 1997, accounting for one-third of all measles cases globally in 2024 [10].

In Croatia, measles vaccination was first introduced in the national childhood vaccination schedule in 1968 and was replaced by the combined measles–mumps–rubella (MMR) vaccine in 1976. The MMR vaccine is mandatory, and the first dose is scheduled at 12–18 months of age, followed by a second dose administered at enrollment into primary school. In the pre-vaccination period, 5000–20,000 cases of measles and rubella were reported each year. In the past two decades, based on data from the Department of Communicable Disease Epidemiology, Croatian Institute of Public Health (CIPH), less than 10 measles cases have been reported annually, except for six import-related outbreaks (2003/2004, 2008, 2014/2015, 2018, 2019, and 2024). Since 2002, MMR vaccination coverage has been above 94% for the first dose and above 98% for the second dose. However, vaccination coverage has been declining since 2012, reaching 90% for the first dose and 89% for the second in 2024 (CIPH data).

Only a few studies in Croatia have assessed the immunity to measles. In a cross-sectional study conducted in 2002, 89.8% of the Croatian residents were IgG-positive to MV, with the highest level of seronegativity (21.4%) observed in one-year-olds. By around one year of age, children no longer have transplacentally derived maternal antibodies. At the same time, they may not yet have received their first measles vaccination, which is typically administered between 12 and 18 months [11]. A more recent regional study (2024) from one eastern Croatian county (Osijek-Baranja County) has found seroprevalence rates ranging from 73.3% to 77.5%, depending on the age group [12]. However, no studies have analyzed the nationwide MV seroprevalence or trends in immunity in Croatia. This retrospective study aimed to analyze measles immunity in the Croatian population over eleven years (2015–2025).

## 2. Materials and Methods

### 2.1. Study Participants

**Figure 1:**
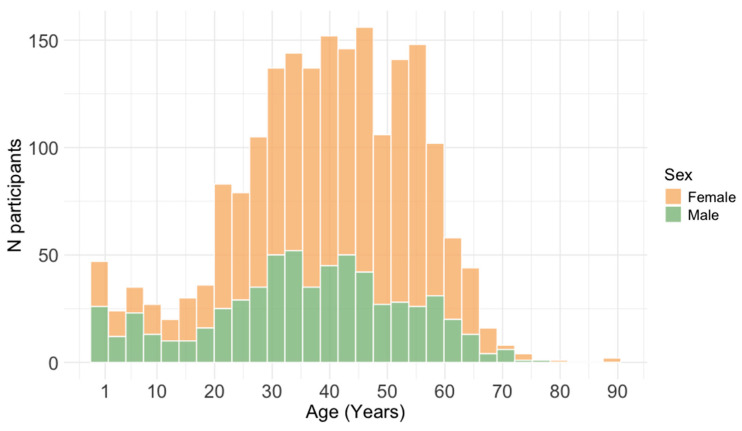
Distribution of study participants by age.

This study included 1998 individuals tested for MV IgG antibodies from January 2015 to December 2025 as part of a routine immunity checkup at the Croatian Institute of Public Health, the largest public health institution in the country. All participants tested during the specified period were included in the study (convenience sample). The exclusion criterion was the presence of a recent febrile illness or rash. Most individuals were tested due to administrative requirements for travel abroad or their interest in seropositivity, triggered by the occurrence of measles in their community or setting. In the tested group, there were 630 (31.7%) males and 1360 (68.3%) females aged 2 months to 89 years (data were missing for 8 patients; Figure 1). The age distribution of study participants by year is presented in Table 1.

For this study, participants were grouped by sex, age (10-year age group), area of residence (continental and coastal), and settlement type (urban and suburban/rural). Suburban settlements were defined as rural areas dominated by agriculture or natural landscapes but located around bigger cities. According to the area of residence, 1212 (60.7%) participants resided in continental and 786 (39.3%) in coastal Croatian regions. According to the settlement type, 1346 (67.4%) participants were residents of urban areas, and 652 (32.6%) were residents of suburban/rural areas.

### 2.2. Serological Testing

MV-specific IgG antibodies were detected using a commercial enzyme immunoassay: Measles ELISA IgG (Virotech Diagnostics, Rüsselsheim, Germany; 2015–2025) or Anti-Measles virus ELISA IgG (Euroimmun, Lübeck, Germany; 2023–2025). The results were calculated and interpreted as follows: Virotech Units (VE) < 9 negative, 9–11 borderline, >11 positive; IU/mL (Euroimmun) < 200 negative, 200–275 borderline; and >275 positive. The manufacturers report a diagnostic sensitivity and specificity of 96.8% and >99.8% for Virotech, and 96.8% and 100% for Euroimmun, respectively. Samples with borderline IgG antibodies (*n* = 52) were retested and excluded from the study.

### 2.3. Statistical Analysis

Descriptive statistics were used to summarize the study population by year, age, sex, geographic region, and settlement type. Age was analyzed as a continuous variable and grouped into predefined 10-year age categories. MV IgG seroprevalence was reported as percentages with corresponding 95% confidence intervals (CI). Annual prevalence estimates and prevalence stratified by broader study periods, age group, region, and settlement type, were visualized using bar plots with 95% CIs. Age distributions according to MV IgG serostatus were summarized using medians and interquartile ranges and visualized with violin plots. Differences in IgG seroprevalence across age groups within each year and within aggregated study periods were assessed using Pearson’s chi-square and Fisher’s exact test. Seroprevalence was visualized by year-by-region heat maps and sex–age population pyramids with the seropositive proportion highlighted within each age–sex stratum. Logistic regression was run on IgG serostatus as the binary outcome and year, age, geographic region, and settlement type as predictors. Regression coefficients are visualized as log-odds estimates with corresponding 95% CIs on effect size plots. A logistic regression model with year as a continuous predictor was used to assess the overall temporal trends in IgG seropositivity, where observed yearly prevalence estimates with 95% CIs were plotted alongside model-based predicted probabilities and corresponding 95% confidence bands. All analyses were two-sided, and significance was reported at the 0.05 level. Statistical analyses were performed in R (version 4.5.2; R Foundation for Statistical Computing, Vienna, Austria), and figures were generated using the ‘ggplot2’ package.

## 3. Results

### 3.1. Temporal Trends in Measles IgG Seropositivity

**Figure 2:**
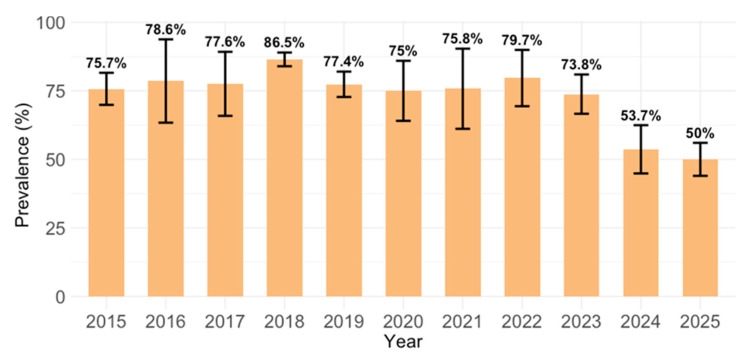
Measles IgG seroprevalence by year (% positive with 95% confidence interval; CI).

MV IgG antibodies were detected in 1501/1998 (75.1; 95% CI = 73.2–77.0) of the serum samples. Analyzing yearly seroprevalence rates, significant differences (*p* < 0.001) in seropositivity were observed between years, ranging from 50.0% (95% CI = 43.8–56.2; 2025) to 86.5% (95% CI = 83.7–88.9; 2018). Lower seroprevalence rates were observed in 2024 (53.7%; 95% CI = 44.4–62.9) and 2025 (50.0%; 95% CI = 43.8–56.2; Figure 2). No difference in the seropositivity rates was observed using the Virotech and Euroimmun ELISA test (2023–2025, Table 2).

### 3.2. Age and Sex-Related Patterns of Measles IgG Seropositivity

**Figure 3:**
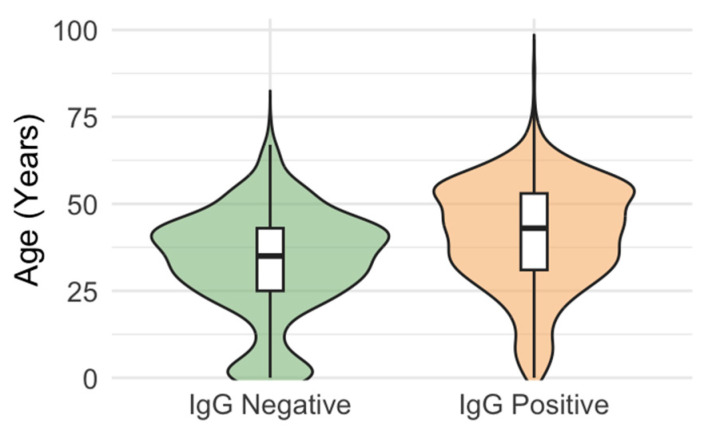
Age distribution of measles IgG seropositive and seronegative individuals. The width of the violin represents the frequency of seropositivity at a given age. A wider violin at older age indicates that more positive individuals are in older age groups, and a shift in the median indicates that older individuals are more likely to be seropositive.

According to age groups, 26.3% of participants under 1 year of age showed transplacentally derived maternal antibodies. The seropositivity was higher in children aged 1–6 months (4/9 positive) than in children aged 7–11 months (1/10 positive). The seroprevalence rates in individuals aged 1–50 years ranged from 60.9 to 77.6%, then increased to 90.1–91.1% in participants older than 50 years (*p* < 0.001; Table 3). IgG-positive individuals were significantly older (*p* < 0.001; median age 43 years, IQR = 31–53) than IgG-negative individuals (median age 35 years, IQR = 25–43; Figure 3).

**Figure 4:**
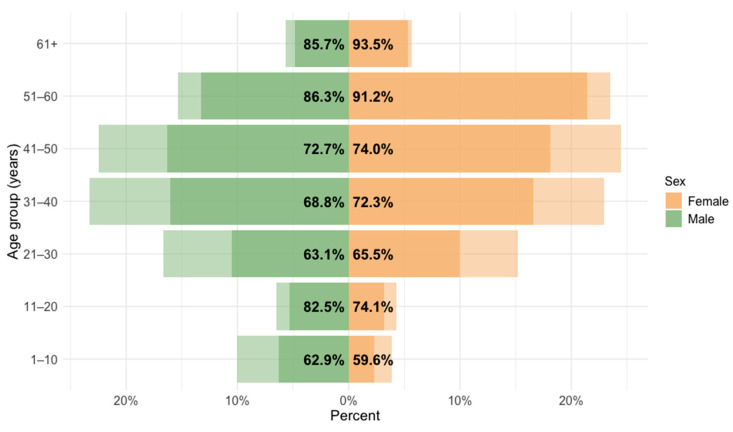
Measles IgG seroprevalence by sex. This sex–age pyramid reflects the structure of the tested population (N participants), with the darker parts of each bar visually displaying the proportion of positive samples within each sex and age category. The exact proportions are indicated by the percentages within each bar.

The MV seroprevalence by sex and age is presented in Figure 4. Due to transplacentally derived maternal antibodies, the age group ≤ 1 year was excluded from the analysis (*n* = 19). Males were more often seropositive (449/627; 71.6%, 95% CI = 70.4–77.5) than females (974/1353; 71.9%, 95% CI = 68.5–73.3); however, these differences were not significant (*p* = 0.173). No significant differences in the prevalence of MV antibodies between sexes were observed in any age group (Supplementary Table S1).

### 3.3. Spatial Patterns of Measles IgG Seropositivity

**Figure 5:**
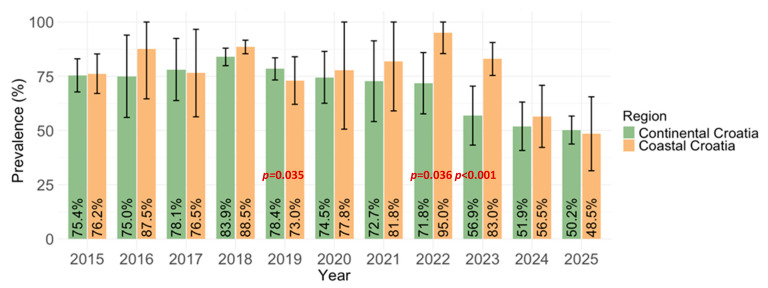
Yearly measles IgG seroprevalence by region (% positive with 95% confidence interval; CI).

Significant differences (*p* < 0.001) in the overall MV seroprevalence were observed between regions. Seropositivity was higher in participants from coastal regions (639/786; 81.3%; 95% CI = 78.5–84.0) than in participants from continental regions (861/1212; 71.0%, 95% CI = 68.4–73.5) (Figure 5).

**Figure 6:**
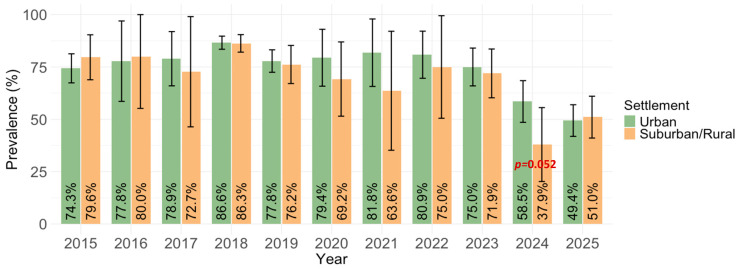
Yearly measles IgG seroprevalence by settlement type (% positive with 95% confidence interval; CI).

Settlement was not a significant predictor of seropositivity (*p* = 0.543). The overall seropositivity among participants living in urban areas was 75.6% (1017/1346; 95% CI= 73.2–77.8%), compared with 74.2% (484/652; 95% CI = 70.7–77.4%) among those residing in suburban or rural areas (Figure 6).

### 3.4. Risk Analysis for Measles Virus IgG Seropositivity

A logistic regression model was used to evaluate the association between different variables (year of sampling, age, region, and settlement type) and MV IgG seropositivity (Table 4). In the multivariable logistic regression, year, age, and region were significantly associated with IgG seropositivity, while settlement was not. Each additional calendar year was associated with lower odds of IgG positivity (OR = 0.82, 95% CI 0.79–0.85, *p* < 0.001); equivalently, the odds were about 18% lower per year ($e^{-0.2009}=0.82$). Each additional year of age was associated with higher odds (OR =1.04, 95% CI = 1.04–1.05, *p* < 0.001); that is, the odds were about 4.3% higher per year of age ($e^{0.0419}=1.04$). Region was also significant (OR = 1.65, 95% CI = 1.30–2.08, *p* < 0.001), indicating about 65% higher adjusted odds in coastal Croatia ($e^{0.4979}=1.65$), while settlement was not significantly associated with IgG seropositivity after adjustment (OR = 1.12, 95% CI = 0.89–1.41, *p* = 0.342).

**Figure 7:**
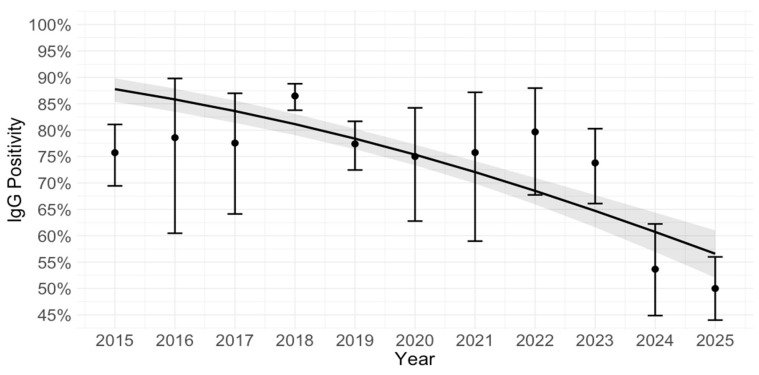
Measles virus IgG positivity over time: observed vs. logistic regression. Each point represents the observed prevalence of IgG positivity in a given year, with 95% confidence intervals (Wilson method) indicated by the error bars. The solid (fitted) line represents the predicted probability of IgG positivity as a smooth function of year, estimated by the logistic regression model. The shaded area around the line shows the 95% confidence interval for the model’s predicted prevalence.

The observed vs. logistic regression for MV IgG positivity over time is presented in Figure 7. The logistic regression plot shows an overall declining trend in IgG seropositivity over the observed period, as reflected by the negative slope of the fitted curve. However, the observed prevalences were relatively stable from 2015 to 2023, fluctuating around a high prevalence, and with overlapping confidence intervals. A more pronounced decline was seen only in 2024 and 2025. Thus, the model captures a general negative temporal association, but the observed data suggest that this declining trend was driven largely by the lower prevalences in the last two years rather than by a steady year-by-year decrease across the entire period.

**Figure 8:**
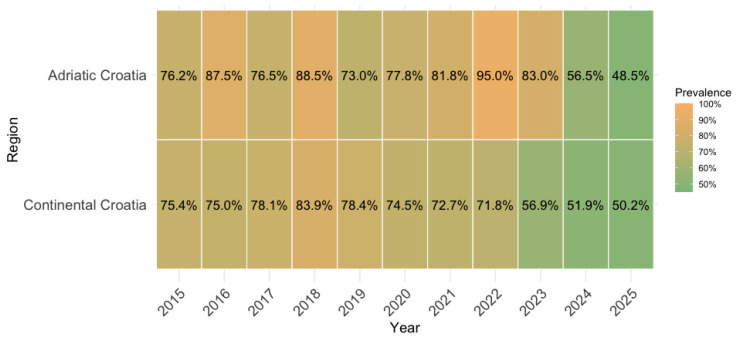
Heatmap of measles IgG seroprevalence by region and year.

Figure 8 presents a descriptive heatmap illustrating MV IgG prevalence across regions and years. A general decreasing trend in prevalence was observed in both regions. On average, we can observe a lower prevalence in continental Croatia, although the lowest overall predicted prevalence was observed in 2025 in coastal Croatia (48.5%). However, the highest observed prevalence was also observed in coastal Croatia in 2022 (95.0%). These results indicate a higher oscillation in the observed prevalence throughout the observed period in coastal Croatia than in continental Croatia.

## 4. Discussion

According to the European Centre for Disease Prevention and Control (ECDC) annual measles surveillance report for 2024, measles vaccination coverage remains below the recommended 95% level in many European Union/European Economic Area (EU/EEA) countries, which is necessary to achieve and sustain measles elimination. In 2023, population-weighted vaccination coverage across EU/EEA countries was 93.9% for the first MV-containing vaccine dose and 88.8% for the second dose, reflecting a slight decline compared with preceding years. Vaccination coverage changed modestly between 2020 and 2023. Sixteen countries showed a decline in coverage for at least one dose, with larger reductions observed for the second dose. Overall, the average vaccination coverage across the EU/EEA decreased slightly, by 0.5% for dose one and 0.8% for dose two in 2023 compared with 2020 [13]. In Croatia, a similar trend is observed. Primary MMR vaccination coverage in 2024 was 90.05%, which was slightly lower than the coverage achieved in 2023 (90.4%) but higher than the coverage recorded in 2022 (89.9%) and 2021 (89.3%). Coverage levels achieved in 2020 (91.2%) and 2019 (93%) were somewhat higher. MMR revaccination at enrollment into primary school reached 89.4% coverage in 2024, which was slightly lower than the coverage levels recorded in 2023, 2022, and 2021 (90.05%, 90.4%, and 90.3%, respectively (CIPH data).

In the present study, the overall MV seroprevalence rate was 75.1%, which is similar to the rate reported in a recent regional study from eastern Croatia (73.3% to 77.5%) [12] but lower than in 2002 (89.8%) [11]. Such a seroprevalence rate is lower than one would expect based on immunization coverage in the last few decades. However, it is important to note that the individuals tested in our study may not fully represent the broader population. Namely, persons who needed serological evidence of MV immunity (mostly travelers who needed documentation of immunity for administrative purposes and members of communities or settings in which measles cases have occurred) are less likely to have evidence of receiving measles-containing vaccines than individuals who do not require testing. Therefore, the results obtained on our sample may underestimate the real seroprevalence in the general population. In Croatia, temporal MV IgG seroprevalence rates have been declining since 2022. Comparing yearly seropositivity in the analyzed period, seroprevalence rates ranged from 75.0% (2020) to 86.5% (2018), while lower seroprevalence rates were observed from 2023 to 2025 (73.8%, 53.7%, and 50.0%, respectively), with an increasing accumulation of susceptible individuals. This result is surprising, since the age structure and other characteristics of the study subjects did not differ significantly in 2024 and 2025 compared to earlier years. Such a large and sudden decrease in seroprevalence cannot be explained by waning immunity either. One of the possible explanations for this observation may be the fact that in late 2024 and early 2025, a small cluster of import-related measles in a health-care setting prompted many health-care workers to check their MV immunity status. As a result, the tested group was not representative of the overall population, but rather biased toward individuals more likely to lack evidence of immunity, which may have led to an apparent underestimation of vaccination coverage. However, a longer follow-up of seroprevalence trends and exploration of other contributing factors is needed to understand the observed results.

Growing vaccine hesitancy or refusal due to perceived low disease risk, mistrust in experts, safety concerns, or misinformation has contributed to fewer people receiving recommended doses [14]. Given that measles elimination strategies typically require at least 95% population immunity to maintain herd protection, the observed levels in 2023–2025 fall considerably below the threshold necessary to prevent sustained transmission. These findings raise concerns regarding the potential re-establishment of endemic measles transmission in the absence of timely and effective public health interventions.

When comparing seroprevalence in the Croatian population with that of other European countries, temporal differences were identified, particularly among individuals born before the introduction of the measles vaccine and those born during the vaccination era.

A study conducted in Madrid, Spain (2007–2010), among young adults reported an MV seroprevalence of 92.1%. The seroprevalence differed among age groups, with significantly higher seroprevalence rates in cohorts born between 1981 and 1986 compared to those born after 1986 [15]. A large cross-sectional study from the adult population in Germany (2008–2011) found that measles IgG seroprevalence exceeded 97% in adults born before 1965, but was under 90% in those born later (89.0% born between 1965 and 1969, 82.0% born between 1970 and 1974 and 74.4–75.8% born 1975 or later), indicating lower immunity among post-vaccination cohorts and potential immunity gaps in younger adults [16]. In a Greek national survey conducted after the 2017–2018 outbreak, the overall seroprevalence was 89.6%. Higher immunity rates and antibody concentrations were observed in individuals aged ≥41 years (94.9%) compared with younger age groups (1–40 years; 83.4%) [17]. In Slovakia, the MV seroprevalence was 91.7% in 2018. Individuals in vaccinated cohorts had significantly lower seropositivity than those with presumed naturally acquired infection [18]. A recent study from Italy (Apulia region, 2023–2024) reported an overall seroprevalence of 85.3%. The seropositivity varied significantly with age, ranging from 40.2% of individuals aged 18–24 years to 96.0% in the 45–54 age group [19].

Our study showed significant age-related increases in measles immunity, too. An increase in the seroprevalence was observed from 60.9% in the 1–10 age group to 77.6% in the 11–20 age group. Thereafter, the seroprevalence rates were stable (65.1–73.7%), followed by a further increase to 90.1–92.3% in individuals older than 51 years. Higher MV seroprevalence among older individuals is largely due to natural infection and repeated immune boosting before the introduction of widespread vaccination, whereas immunity in younger populations relies primarily on vaccination and may decline in the absence of ongoing viral circulation. Older cohorts were more likely to have been exposed to wild-type MV during childhood, particularly those born before routine immunization programs were implemented. Natural infection typically induces stronger and longer-lasting immunity compared with vaccine-induced protection, resulting in higher and more persistent antibody levels. Furthermore, during the pre-elimination era, widespread circulation of measles led to repeated exposures in older cohorts, further enhancing antibody titers over time. In a group of children younger than one-year, transplacentally derived maternal antibodies were more frequently detected in those aged less than six months.

In one more recent Italian study conducted between 2019 and 2020, a U-shaped seroprevalence curve was observed. Seroprevalence declined markedly from 94.2% in children aged 6–9 years to lower levels in the subsequent age groups (89.5% in 10–14 years, 89.7% in 15–19 years, and 89.3% in 20–39 years) and increased significantly thereafter to 97.6% among older adults [20]. Similarly, a 2024 Serbian study involving populations from Belgrade reported high seropositivity rates in both children (90.7%) and older adults (90.7% and 98.4%, respectively), whereas individuals aged 30–49 showed significantly lower IgG levels [21].

There was no significant difference in seropositivity between males and females in Croatia. Given the mandatory vaccination policy, comparable seropositivity rates between males and females would be expected.

Our study revealed significant regional differences in MV seropositivity, with higher seroprevalence rates in coastal regions (81.4%) compared to continental regions (71.0%).

Coastal Croatian regions are typically more integrated into international mobility networks due to tourism and seasonal labor migration, resulting in a higher rate of MV importation compared with continental regions. From a transmission-dynamics perspective, repeated viral introductions increase the local force of infection, even in settings without sustained endemic circulation. In populations with existing immunity gaps, stochastic importations may initiate short transmission chains that either result in naturally acquired immunity among susceptible individuals or prompt reactive public health interventions, such as targeted MMR catch-up campaigns. Additionally, secondary immune boosting in previously vaccinated individuals following exposure may increase detectable antibody titers in serosurveys. Together, elevated importation pressure, episodic transmission, and intensified immunization responses can lead to higher seroprevalence in highly connected coastal regions relative to more demographically stable inland areas. In some coastal regions, there may historically have been better local engagement with vaccination or more aggressive catch-up campaigns when measles cases were detected. Conversely, inland areas with pockets of vaccine hesitancy or lower healthcare access might have lower seroprevalence because fewer individuals received or completed the recommended two-dose MMR schedule.

In the post-elimination period, Croatia has predominantly detected globally circulating genotypes B3 and D8, consistent with wider European trends. For example, molecular analysis of the 2019 Split–Dalmatia outbreak identified both B3 and D8 strains, including multiple sequence variants, confirming repeated importations and limited local clustering [22]. These genotypes are now the main lineages responsible for outbreaks across Europe and globally, having replaced earlier endemic genotypes [23].

We found no significant difference in the MV seroprevalence between residents of urban and suburban/rural regions. These results can be explained by the high degree of centralization and uniformity of the national immunization program, which ensures comparable MMR vaccine access and coverage across geographic settings. When vaccination delivery is systematically organized through primary healthcare networks with standardized schedules and high overall uptake, spatial heterogeneity in immunity is minimized. Additionally, frequent population mixing between urban and rural areas through commuting, education, and social networks reduces epidemiological isolation and promotes homogenization of immunity profiles. In a context where endemic measles transmission has been interrupted, and immunity is primarily vaccine-derived rather than infection-driven, seroprevalence patterns reflect vaccination coverage rather than population density; thus, similar coverage levels naturally produce similar serological immunity in both urban and rural populations.

There are some limitations of this study that need to be addressed. The use of a convenience sample (individuals tested routinely for measles immunity) may not be representative of the broader Croatian population. Sampling individuals preparing for international travel or from communities concerned about recent measles outbreaks may introduce demographic bias. Travelers often have higher socioeconomic status and better healthcare access, which could lead to higher observed seropositivity, while outbreak-affected communities may include under-vaccinated or vaccine-hesitant groups, showing lower immunity. This mixed sampling may therefore over- or underestimate true population immunity. Additionally, two tests (Virotech and Euroimmun) were used for serological testing in the period 2023–2025. However, comparison of the results showed no difference in seropositivity between the two ELISA assays, indicating that the observed lower seroprevalence was not related to the testing method.

## 5. Conclusions

While historical coverage with the MMR vaccine in Croatia is high, targeted testing during recent outbreaks has identified vulnerable groups (such as healthcare workers), highlighting the need for targeted catch-up campaigns. Beyond access issues, the COVID-19 pandemic may have amplified hesitancy through factors such as increased exposure to misinformation, declining trust in public health institutions, and heightened skepticism toward vaccines in general. If a downward seroprevalence trend persists, Croatia may face an increased risk of measles outbreaks, especially in communities with lower vaccination coverage. Strengthening surveillance systems, improving vaccination uptake, implementing catch-up immunization campaigns, and addressing vaccine hesitancy through targeted public health communication strategies will be critical to restoring herd immunity and preventing re-establishment of endemic transmission. Further studies are warranted to identify specific demographic groups contributing to declining immunity and to guide evidence-based intervention strategies.

## Figures and Tables

**Table 1:** Age structure of study participants by year.

Year	Years						Outliers
	Min	Q1	Median	Q3	Max	IQR	
2015	3 months	14	31	41.8	67	27.8	0
2016	5 months	22.8	34.5	41.0	62	18.2	0
2017	7 months	22	33	44	73	22	0
2018	2 months	31	42	52	89	21	1
2019	1	30.2	39	51	88	20.8	1
2020	10	30.8	40.5	48.5	69	17.8	0
2021	3	31.0	47	53	69	22	0
2022	5	31.5	42	52	67	20.5	0
2023	4 months	34	45	56	81	22	1
2024	1	30.5	41	52.5	73	22	0
2025	1	31	42.5	49	72	18	2

Q1 = first quartile; Q3 = Third quartile; IQR = Interquartile range.

**Table 2:** Measles IgG seroprevalence results tested by different ELISA tests (2023–2025).

Year	Virotech			Euroimmun			Total		
	N Tested	N (%) MV IgG	95% CI	N Tested	N (%) MV IgG	95% CI	N Tested	N (%) MV IgG	95% CI
2023	100	74 (74.0)	64.3–82.3	45	33 (73.3)	58.1–85.4	145	107 (73.4)	65.8–80.7
2024	57	31 (54.4)	40.7–67.4	66	35 (53.0)	40.3–65.4	123	66 (53.7)	44.4–62.7
2025	90	44 (48.9)	38.2–59.6	174	88 (50.6)	42.9–58.2	264	132 (50.0)	43.8–56.2

MV = Measles virus; CI = Confidence interval.

**Table 3:** Measles IgG seroprevalence by age.

Age Group	Tested N (%)	IgG Positive N (%)	95% CI	*p*
<1 year	19 (0.9)	5 (26.3%)	11.8–48.8	<0.001
1–10 years	115 (5.7)	70 (60.9)	51.7–69.3	
11–20 years	98 (4.9)	76 (77.6)	68.3–84.7	
21–30 years	312 (15.6)	203 (65.1)	59.6–70.1	
31–40 years	456 (22.8)	324 (71.1)	66.7–75.0	
41–50 years	471 (23.6)	347 (73.7)	69.5–77.4	
51–60 years	415 (20.8)	374 (90.1)	86.9–92.6	
61+ years	112 (5.5)	102 (91.1)	84.2–95.6	

**Table 4:** Estimated risk for measles IgG positivity.

Parameter	Estimated Risk (Log Odds)	Standard Error	*p*
Year	−0.200	0.017	<0.001
Age	0.041	0.003	<0.001
Region	0.497	0.120	<0.001
Settlement	0.112	0.118	0.342

## Data Availability

The original contributions presented in the study are included in the article; further inquiries can be directed to the corresponding author.

## Supplementary Materials

The following supporting information can be downloaded at: https://www.mdpi.com/article/10.3390/vaccines14050393/s1, Table S1: Measles IgG seroprevalence by sex and age.

## Abbreviations

The following abbreviations are used in this manuscript:

ICTV	International Committee on Taxonomy of Viruses
MV	Measles virus
MMR	Measles–Mumps–Rubella
CIPH	Croatian Institute of Public Health
ECDC	European Centre for Disease Prevention and Control
EU/EEA	European Union/European Economic Area
