# Supplementary material for: Assessment of Measles Immunity in the Croatian Population: A Retrospective Seroprevalence Study, 2015–2025
Source: Vaccines (Basel). 2026 Apr 27;14(5):393. doi: 10.3390/vaccines14050393 (PMC13211742; doi:10.3390/vaccines14050393)
Supplement: Supplementary file 1 [file vaccines-14-00393-s001.zip › vaccines-4229678-supplementary.pdf]

Supplemental Table S1. Measles IgG seroprevalence by sex and age.

Age group (Years)	Male			Female			
	**N Tested**	**N (%) MV IgG positive**	**95%CI**	**N Tested**	**N (%) MV IgG positive**	**95%CI**	***p***
1–10	62	39 (62.9)	50.5–73.8	52	31 (59.6)	46.1–71.8	0.847
11–20	40	33 (82.5)	68.1–91.3	58	43 (74.1)	61.6–83.7	0.460
21–30	103	65 (63.1)	53.5–71.7	206	135 (65.5)	58.8–71.7	0.705
31–40	144	99 (68.8)	60.8–75.8	310	224 (72.2)	67.0–76.9	0.438
41–50	139	101 (72.7)	64.7–79.4	332	245 (73.0)	69.0–78.4	0.818
51–60	95	82 (86.3)	78.0–91.8	318	290 (91.2)	87.6–93.8	0.173
61+	35	30 (85.7)	69.7–95.2	77	72 (93.5)	85.5–97.8	0.180

*For 8 participants, data on sex are missing
